# Mitochondrial Dysfunction: A Critical Link Between Maternal Diet and Offspring Metabolic Health

**DOI:** 10.3390/biom16081106

**Published:** 2026-07-29

**Authors:** Chuhan Shao, Hanmo Lin, Jie Yu, Haiyan Chen, Yaolin Ren, Jing Ren, Yuan Zeng, Yifan Wu, Qian Zhang, Xinhua Xiao

**Affiliations:** NHC Key Laboratory of Endocrinology (Peking Union Medical College Hospital), Diabetes Research Center of Chinese Academy of Medical Sciences, Department of Endocrinology, Peking Union Medical College Hospital, Chinese Academy of Medical Sciences & Peking Union Medical College, Beijing 100730, China; sch999@student.pumc.edu.cn (C.S.); s2025001030@student.pumc.edu.cn (H.L.); b2023001044@student.pumc.edu.cn (J.Y.); s2024001029@student.pumc.edu.cn (H.C.); s2023001083@pumc.edu.cn (Y.R.); renjing@student.pumc.edu.cn (J.R.); b2024001052@student.pumc.edu.cn (Y.Z.); pumc_wuyifan@student.pumc.edu.cn (Y.W.)

**Keywords:** maternal, overnutrition, undernutrition, mitochondria, offspring metabolism

## Abstract

**Background/Objectives:** The developmental origins of health and disease (DOHaD) theory suggests that intrauterine and early postnatal life represents a critical window for programming lifelong health trajectories and disease susceptibility in offspring. Maternal nutritional imbalance during this period is closely associated with obstetric complications and an elevated risk of metabolic disorders in children. As central metabolic hubs, mitochondria constitute a critical axis linking adverse in utero exposure to metabolic defects in offspring across generations. **Methods:** In this narrative review, we searched PubMed and Web of Science (up to 8 July 2026) for English-language literature linking maternal metabolic conditions and mitochondrial dysfunction. We included in vivo, in vitro, and clinical studies, explicitly excluding primary inherited mtDNA mutations and nonnutritional toxicant exposures to isolate nutritional programming effects. **Results:** Maternal metabolic stress induces multifaceted, tissue-specific mitochondrial alterations in the developing offspring. Rather than a uniform systemic decline, mitochondrial reprogramming exhibits profound spatial and cellular heterogeneity across critical metabolic organs, including the placenta, liver, skeletal muscle, heart, and hypothalamus. These developmental adaptations often manifest as molecular compensations, such as altered mitochondrial dynamics, perturbed biogenesis, and shifted OXPHOS capacity, ultimately leading to functional bioenergetic failure, oxidative stress, and the establishment of insulin resistance. **Discussion:** Organ-specific mitochondrial dysfunction drives the maternal transmission of metabolic syndrome. Targeting these mechanisms via dietary modifications, exercise, pharmacological agents, and mitochondrial transplantation offers promising strategies to rescue bioenergetics and prevent metabolic diseases in offspring.

## 1. Introduction

Maternal lifestyle during gestation and lactation affects newborn metabolism, establishing lifelong effects on metabolic health. This concept is firmly rooted in the developmental origins of health and disease (DOHaD) paradigm [1], which evolved from the Fetal Origins of Adult Disease (FOAD) hypothesis initially conceptualized by David Barker [2,3]. Barker and colleagues first traced this link by revealing that regions with high rates of low birth weight paradoxically suffered from increased adult mortality due to cardiometabolic diseases [4]. Barker’s pioneering epidemiological observations, alongside the thrifty phenotype hypothesis proposed with Hales, established that adverse intrauterine environments program susceptibility to cardiometabolic disorders later in life [5]. Crucially, specific maternal risk factors including nutritional status or obstetric complications like maternal prepregnancy overweight or obesity [6], abnormal gestational weight gain (GWG) [7], gestational diabetes mellitus (GDM) [8] and preeclampsia [9], suboptimal nutritional habits during pregnancy [10], and a lack of exclusive breastfeeding [11] are all major determinants of adverse metabolic programming.

Among these factors, this review focuses on maternal overnutrition or malnutrition, as well as associated metabolic disorders such as GDM, which can profoundly compromise offspring metabolic health [12,13,14]. In both clinical observations and experimental models, maternal obesity or maternal overnutrition increases offspring susceptibility to metabolic syndrome, type 2 diabetes, obesity, and cardiovascular disease in adulthood [15]. Conversely, maternal nutrient restriction during pregnancy frequently results in intrauterine growth restriction (IUGR) or small-for-gestational-age (SGA) offspring, a situation associated with increased lifelong risks of cardiometabolic disorders. Among the various forms of maternal macronutrient and micronutrient deficiencies, protein restriction (PR) during pregnancy remains among the most prevalent and well-studied insults globally, particularly in low- and middle-income countries, as well as in subpopulations with poor dietary diversity [16]. Maternal nutritional status affects metabolic health in offspring [17]. Body mass index (BMI) is a commonly used standard for measuring maternal obesity or undernutrition. According to a Lancet report, low maternal BMI is associated with IUGR. Undernutrition among mothers and children has long-term consequences for the risk of metabolic and cardiovascular diseases [18,19]. Currently, diet patterns tend to move towards Western-style high-sugar, high-fat, high-energy-density, and low-fiber diets, which has led to a significant increase in the global prevalence of metabolic diseases among women of childbearing age [17]. Recent meta-analyses and umbrella reviews have indicated that children born to mothers with obesity or excessive gestational weight gain face an elevated risk of developing overweight and obesity, with the most pronounced effects observed during late childhood [20]. Other evidence shows that maternal adiposity increases offspring body fat percentage and fat mass [21]. Moreover, fetal exposure to GDM is associated with significantly increased risks of overweight and obesity in offspring, with higher triglyceride levels, low-density lipoprotein cholesterol (LDL-c) levels, and blood pressure [22].

Recent evidence has suggested that epigenetic changes, gut dysbiosis, and placental dysfunction mechanisms are involved in transgenerational effects [17,23]. To be specific, early life environment affects offspring epigenetic modifications, including DNA methylation, histone modification, and microRNAs, to affect gene expression levels [24]. Gut environment is established early in life, with the composition of maternal aberrant gut microbiota influenced by nutritional status, affecting gut dysbiosis in the offspring [25]. Placental dysfunction, characterized by inflammation and oxidative stress, threatens the fetus during obese pregnancy [26]. Notably, emerging evidence indicates that mitochondrial dysregulation is a transgenerational, ever-increasing risk factor for metabolic disorders following maternal nutritional imbalance [13].

Mitochondria are the powerhouse of the cell, mediating metabolism and acting as a central environmental sensor orchestrating environmental exposures and nuclear DNA (nDNA) and mitochondrial DNA (mtDNA) responses, representing their ability to function as biosynthetic and signaling hubs [27,28]. Mitochondria are maternally inherited, and an aberrant maternal metabolic environment might induce the formation of dysfunctional mitochondria or altered mtDNA/nDNA in the oocyte, which are subsequently transmitted to the offspring via the maternal lineage in both human and animal studies [29]. This mitochondrial cross-generational transmission contributes to the pathogenesis of metabolic dysregulation in various offspring organs through impaired oxidative phosphorylation (OXPHOS) and energy homeostasis, excessive reactive oxygen species (ROS) production and oxidative stress, and dysregulated mitochondrial fusion/fission, mitophagy, and mitochondrial quality control.

Since David Barker initially conceptualized the DOHaD hypothesis, extensive research has established the link between early-life environmental exposures and adult-onset metabolic diseases. However, despite the growing recognition of mitochondria as the central hub in this transgenerational programming, a significant literature gap remains. Most existing reviews either focus exclusively on a single tissue or broadly discuss systemic epigenetic changes, lacking a comprehensive synthesis of how mitochondrial functional adaptations and maladaptations differ distinctly across various offspring organs. Furthermore, there is a scarcity of integrated analyses evaluating how these organ-specific mitochondrial defects can be strategically targeted by emerging therapies, particularly novel interventions like mitochondrial transplantation.

Therefore, the specific aim of this review is to provide a clear and up-to-date perspective on metabolic changes related to early health problems triggered by maternal nutritional imbalances, with a central focus on mitochondrial dysfunction. First, we aim to provide an up-to-date synthesis of how maternal nutritional imbalances program organ-specific metabolic alterations. Second, we critically discuss the pathogenic mechanisms through which transgenerational mitochondrial dysfunction drives these diseases. Finally, we review therapeutic approaches targeting mitochondrial dysfunction due to maternal nutritional stress through dietary interventions, pharmacological agents, physical exercise, and mitochondrial transplantation.

By integrating these transgenerational mitochondrial contributions, this review aims to elucidate their roles in metabolic health and reveal therapeutic targets.

## 2. Methods

This work is designed as a narrative review instead of a systematic review, so no formal PRISMA flow diagram, risk-of-bias assessment, GRADE assessment, or quantitative meta-analysis was performed.

A search of the PubMed and Web of Science databases was conducted on 8 July 2026, to identify relevant studies. Our search was restricted to English publications. Our search strategy primarily focused on terms including (“Maternal obesity” OR “Maternal overnutrition” OR “Maternal malnutrition” OR “Maternal undernutrition” OR “gestational diabetes” OR (“obesity” AND “pregnant”) OR (“nutrition” AND “pregnant”) OR (“protein restriction” AND “pregnant”) OR (“obesity” AND “gestation”) OR (“nutrition” AND “gestation”) OR (“protein restriction” AND “gestation”)) AND (“mitochondria dysfunction” OR “mitophagy” OR “mitochondr*”). To maximize sensitivity and capture early studies, initial searches did not impose strict subject heading restrictions on the target organ or offspring metabolic disorders, and the screening of offspring characteristics focused on the subsequent manual screening stage.

To ensure the relevance and quality of the reviewed literature, specific inclusion and exclusion criteria were applied. Inclusion criteria were as follows: (1) original research (both in vivo animal models and in vitro cell studies), clinical trials, and relevant reviews on maternal transmission of mitochondrial function and metabolic health; (2) articles published in English. The exclusion criteria included: (1) studies focusing on mitochondrial dysfunction induced by primary, inherited mtDNA genetic mutations rather than environmental/nutritional programming; (2) research primarily assessing non-nutritional maternal environmental exposures like heavy metals, smoking, or endocrine disruptors; (3) non-English publications; and (4) records without full-text availability.

In summary, this targeted search strategy ensures that the reviewed literature strictly focuses on mitochondrial dysfunction driven by maternal nutritional imbalances, providing a robust foundation for our subsequent mechanistic discussions.

## 3. Mitochondrial Function in Lipid and Glucose Metabolism

### 3.1. Mitochondrial Quality Control (MQC)

Mitochondria are dynamic organelles involved in multiple processes, such as energy metabolism, redox homeostasis, and cell differentiation [30], and can be modified by nutritional excess or metabolic disorders [31,32], which affect MQC (biogenesis, dynamics, and mitophagy).

Rats fed a high-fat diet (HFD) exhibited downregulated expression of peroxisome proliferator-activated receptor-γ (PPARγ) coactivator 1α (PGC-1α) and reduced mtDNA content. In patients with metabolic dysfunction-associated fatty liver disease (MAFLD), the expression of crucial proteins for mitochondrial biogenesis, PGC-1α, nuclear respiratory factor 1 (NRF1), and transcription factor A, mitochondria (TFAM), is downregulated, leading to decreased mitochondrial biogenesis and reduced mitochondrial quantity and function [33,34]. PGC-1α initiates a transcriptional cascade involving the activation of transcription factors, including NRF1 and NRF2, and culminates in the expression of TFAM, ultimately facilitating mtDNA transcription and replication [28]. Mitochondrial biogenesis influences adipogenesis through closely linked transcription factors. Reduced adipogenesis is associated with local fat accumulation and insulin resistance [31].

Mitochondrial fission and fusion are dynamic processes that shape mitochondrial morphology in response to cellular metabolic demands [28]. Mitochondrial fusion involves the GTPase mitofusin (MFN)1/2 at the outer membrane [35] and optic atrophy 1 (OPA1) at the inner membrane, thereby enabling content exchange and crista stabilization. Fission, conversely, is driven by the recruitment of dynamin-related protein 1 (DRP1) to specific outer membrane receptors, a process that partitions damaged mitochondrial components for degradation [30]. Excess nutrients increase ROS production and fission and inhibit mitophagy [36]. In the liver and skeletal muscle of HFD-fed animals, increased fission and decreased fusion are observed [37]. Deficient fusion or excessive fission impairs metabolic flexibility and intensifies oxidative stress.

Mitophagy is defined as mitochondrial autophagy that removes superfluous or dysfunctional mitochondria, thereby maintaining optimal mitochondrial numbers and intracellular homeostasis [38]. This process is predominantly governed by the PTEN-induced putative kinase 1 (PINK1)–Parkin pathway acting as a key regulator. Efficient removal of damaged mitochondria depends on mitophagy, yet this process is impaired in individuals with obesity and type 2 diabetes, as evidenced by the decreased expression of key mitophagy regulators, including PINK1 and Parkin [39]. Impaired mitophagy is observed in the pathophysiology of metabolic tissues associated with insulin resistance and diabetic complications in animal models. In livers affected by metabolic dysfunction-associated steatohepatitis (MASH), impaired mitophagy and the resulting accumulation of mitochondrial ROS (mROS) lead to the accumulation of dysfunctional mitochondria, thereby promoting lipid deposition [40].

### 3.2. Mitochondrial Metabolism and Oxidative Stress

When MQC is inhibited, structurally compromised mitochondria with diminished membrane potential and damaged mtDNA exhibit an impaired ability to oxidize glucose and fatty acids, leading to reduced mitochondrial respiratory activity and adenosine triphosphate (ATP) synthesis, while the production of ROS that drive metabolic dysfunction is increased [41].

OXPHOS drives mitochondrial ATP synthesis via the catabolism of carbohydrates and fatty acids [30]. However, during this process, electron leakage from the electron transport chain (ETC) and enzymes within the fatty acid oxidation (FAO) pathway simultaneously generate ROS [42,43]. Mitochondrial FAO in adipose tissue is inhibited in obesity as well as in liver tissues affected by MASH. In mice with MASH under conditions of nutrient excess, carnitine palmitoyltransferase 1/2 (CPT1/2) expression is downregulated, limiting the mitochondrial import of long-chain fatty acids [44]. Obese rats exhibit reduced adipose tissue CPT1 mRNA expression [31]. Moreover, MASH downregulates the expression of AMP-activated protein kinase (AMPK), PGC-1α, and peroxisome proliferator-activated receptor α (PPARα) [45]. These proteins are key regulators of FAO that normally induce enzymes such as CPT1 and β-hydroxyacyl-CoA dehydrogenase. Thus, their suppression may contribute to impaired FAO capacity [46]. Glucose oxidation is also reduced, as decreased levels of metabolites related to glucose oxidation in the mitochondrial matrix are observed in high-fat-diet-fed mice [31].

**Figure 1:**
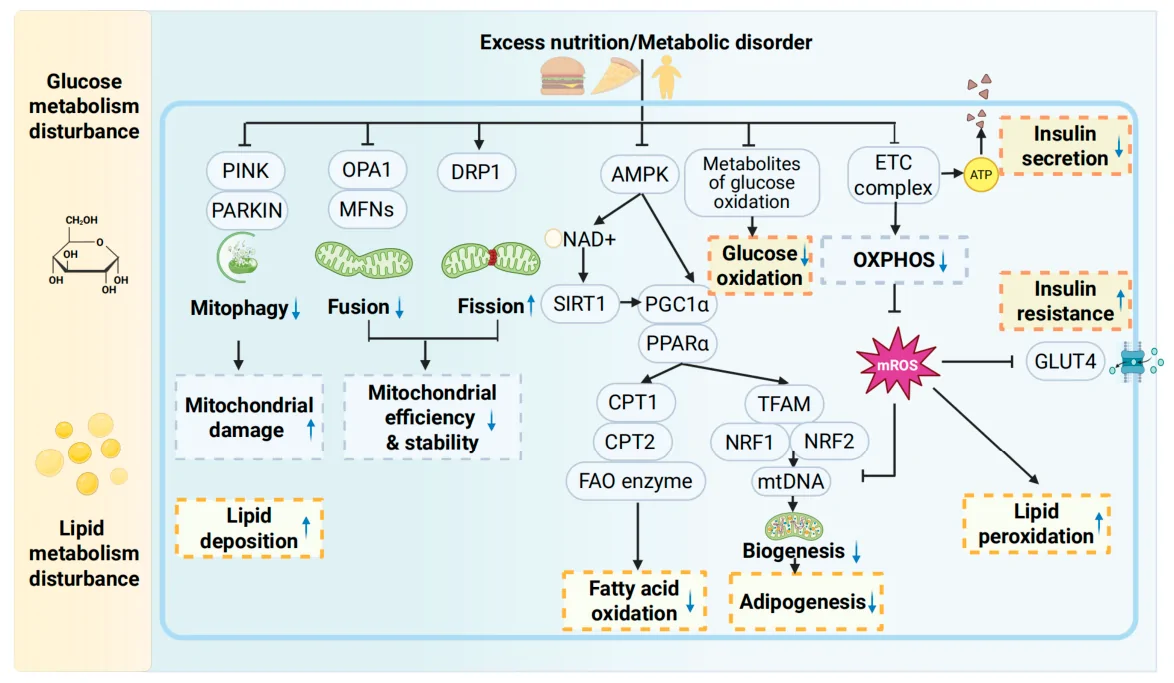
Typical alterations in lipid and glucose metabolism and mitochondrial function in metabolic disorders or exposure to excess nutrition (Created with BioRender.com Chuhan Shao. (2026)). When exposed to excess nutrition or metabolic disorder, fatty acid oxidation is impaired by dysregulated AMPK, PGC-1α, and PPARα, reduced CPT1/2-mediated transport of long-chain fatty acids, and decreased activity of β oxidation enzymes and cofactors. This metabolic deficit, together with ETC dysfunction, lowers OXPHOS and ATP output, increases mROS, and promotes oxidative stress, lipid peroxidation, and insulin resistance. Moreover, mitochondrial integrity is compromised, exhibiting abnormal MQC. The down-regulation of PGC-1α and PPARα inhibits NRF1/2, TFAM and mtDNA, leading to impaired biogenesis and adipogenesis. Disturbance in fission and fusion lead to impaired mitochondrial efficiency and stability. Mitophagy is impaired, leading to lipid deposition. PINK1: PTEN induced kinase 1; OPA1: optic atrophy 1; MFN: mitofusin; DRP1: dynamin-related protein 1; AMPK, AMP-activated protein kinase; SIRT1: Sirtuin 1; PPARα, peroxisome proliferator-activated receptor alpha; CPT1/2: carnitine palmitoyltransferase 1/2; FAO: fatty acid oxidation; PGC-1α: peroxisome proliferator-activated receptor-γ coactivator 1α; NRF: nuclear respiratory factor; TFAM: transcription factor A, mitochondria; mtDNA: mitochondrial DNA; OXPHOS: oxidative phosphorylation; ETC: electron transport chain; mROS: mitochondrial reactive oxygen species; GLUT4: glucose transporter type 4; Mark explanations: an arrow, induction; a T-line, inhibition; upward (↑) and downward (↓) arrows indicate an increase (or upregulation) and a decrease (or downregulation) in the respective processes or levels.

Disruption of FAO creates an imbalance in substrate supply to the ETC, leading to disruption of the ETC and excessive mROS generation. Limited OXPHOS capacity was observed in mice with diet-induced obesity, and reduced levels of OXPHOS-related mitochondrial protein subunits were detected in diabetic mice [31]. Excessive mROS leads to high oxidative stress, which in turn induces redox-related alterations in nucleic acids, proteins, and membranes while impairing mitochondrially encoded subunits of ETC complexes, ultimately leading to decreased mitochondrial ATP production [36] and the formation of a detrimental feedback loop. This decreased ATP production impairs insulin secretion [47]. Increased ROS levels are linked to the development of insulin resistance, which is regulated by glucose transporter type 4 (GLUT4) downregulation [31] and lipid peroxidation [32] (Figure 1).

Overall, excess nutrition and metabolic disorders severely disrupt the delicate balance of mitochondrial quality control and oxidative phosphorylation. The impairment of mitochondrial biogenesis, dynamics, and mitophagy, coupled with compromised ATP synthesis and excessive mROS generation, drives a vicious cycle of oxidative stress, lipid peroxidation, and diminished FAO, which collectively anchor the cellular pathogenesis of insulin resistance and metabolic collapse.

## 4. Maternal Nutritional Imbalance Induces Transgenerational Mitochondrial Dysfunction Through the Germline

Two leading mechanisms have been proposed to explain this transgenerational inheritance: inheritable epigenetic modifications of nuclear genes encoding mitochondrial proteins or direct epigenetic modifications of mtDNA itself [29]. Owing to the maternal mitochondrial inheritance pattern, oocytes are vital for the passage of mitochondrial dysfunction across generations. Oocytes from rodents fed an HFD show mitochondrial disruptions, involving altered mitochondrial membrane potential (MMP), decreased ATP synthesis and FAO, as well as abnormal mtDNA levels and mitochondrial biogenesis [27]. When mice develop diet-induced metabolic syndrome before and throughout gestation, they pass down disrupted mitochondrial dynamics and irregular ETC complex proteins via defective oocytes to the F3 generation, even if the F1 progeny maintain a standard diet [48].

However, additional research has suggested that this mitochondrial dysfunction can be independent of direct maternal mitochondrial inheritance through oocytes. In murine models, mitochondrial defects can be inherited across generations, resulting in impaired cardiac mitochondrial function in offspring. Notably, these abnormalities are transmissible through the paternal germline. Given that mitochondrial inheritance is predominantly maternal, the transmission of cardiac mitochondrial impairments to the F2 generation via the F1 male offspring of obese mothers implies that the transgenerational inheritance of mitochondrial defects in descendants of obese females is through the transmission of excess nutrition-induced effects in the nucleus, not through the direct passage of abnormal mitochondria [49].

Consequently, maternal dietary imbalance can establish transgenerational mitochondrial deficiencies and induce structural and functional changes in various offspring tissues through the germline; however, mitochondrial tracing studies and pronuclear transfer assays are needed to definitively elucidate the underlying mechanism. Obesity in the mother, grandmother, and even great-grandmother in the maternal lineage may determine metabolic disorders in later generations [49].

In conclusion, the transgenerational transmission of metabolic defects is profoundly mediated through the germline. Whether driven by the direct inheritance of compromised mitochondria from oocytes, persistent epigenetic modifications of nuclear-encoded mitochondrial genes, or emerging paternal epigenetic factors, this cross-generational mitochondrial programming establishes the fundamental cellular vulnerability for offspring metabolic diseases.

## 5. Altered Transgenerational Metabolic Changes Through Mitochondrial Function

**Figure 2:**
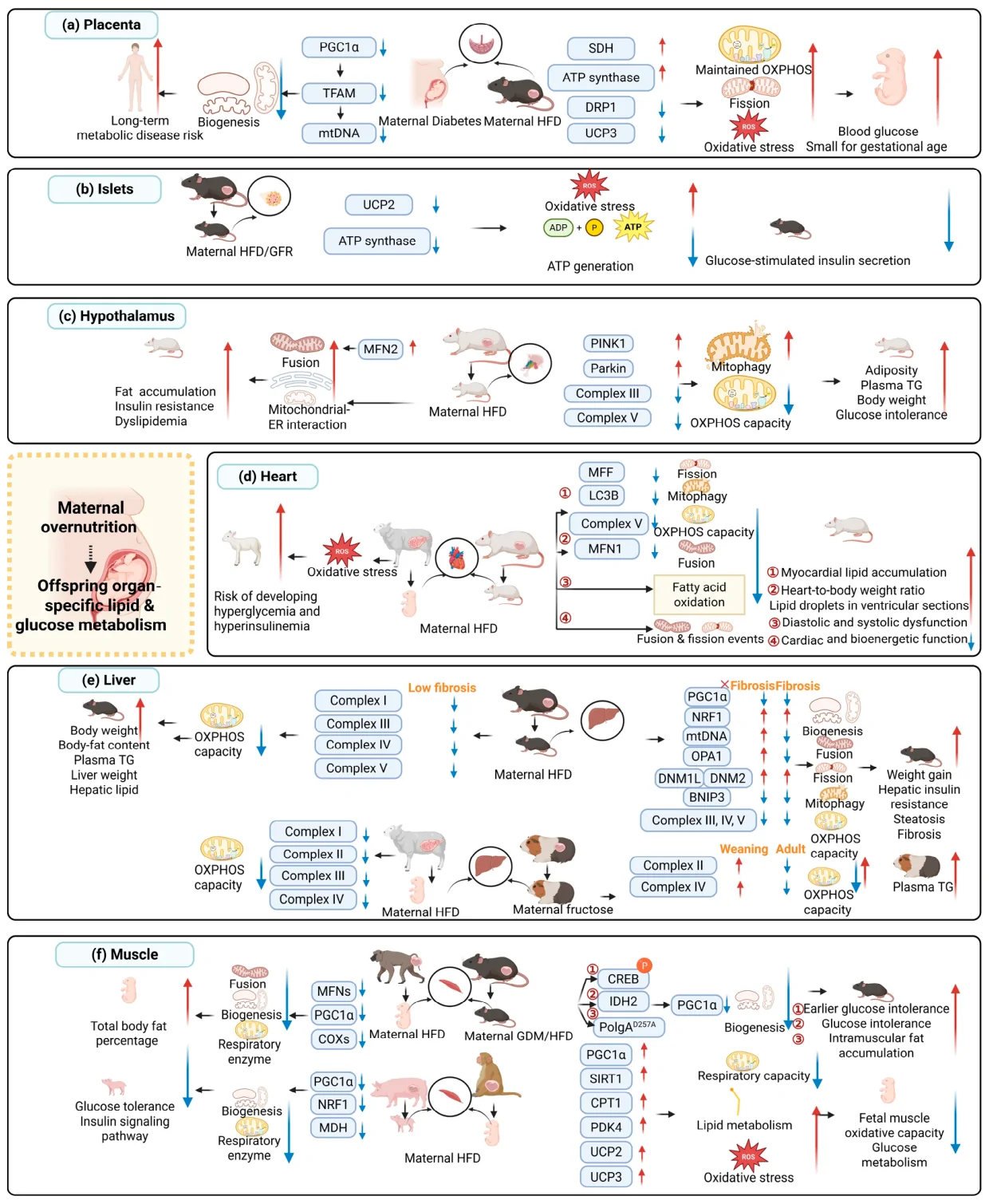
Effects of maternal overnutrition on organ-specific lipid and glucose metabolism disruptions in offspring through mitochondrial dysfunction (Created with BioRender.com. Chuhan Shao. (2026)). (**a**) Mothers with diabetes show a reduction in biogenesis in the placenta, leading to an increased risk of metabolic disease in the long term in humans. In maternal HFD mice, oxidative stress increases and oxidative stress elevates, with maintained OXPHOS, leading to increased SGA risk and blood glucose level. (**b**) Maternal HFD mice showed an increased ROS level as well as a decrease in ATP production, leading to a reduction in glucose-stimulated insulin production in islets. (**c**) In hypothalamus, maternal HFD triggers aberrant mitochondrial remodeling characterized by excessive fusion and mitochondrial–ER interaction, leading to increased fat accumulation, insulin resistance, and dyslipidemia in rats. (**d**) Impaired fission, fusion, mitophagy, OXPHOS, fatty acid oxidation and increased oxidative stress are observed in the heart of maternal HFD offspring in mice and sheep, leading to an increase in myocardial lipid accumulation, heart-to-body weight ratio, and lipid droplets in ventricular sections. (**e**) OXPHOS increases at the early stage of MAFLD but decreases as fibrosis develops in the offspring liver of maternal HFD or fructose. MQC is also diminished in MAFLD in offspring of maternal overnutrition, resulting in weight gain, steatosis, and insulin resistance. (**f**) Maternal overnutrition downregulates offspring muscle biogenesis through CREB phosphorylation, IDH2, and the presence of PolG mutation. Fusion and respiratory enzymes are also reduced, leading to insulin resistance and intramuscular fat accumulation in mice, pigs and baboons. In Japanese macaques, respiratory capacity is reduced, while lipid metabolism and oxidative stress are enhanced. HFD: high-fat diet; PGC-1α: peroxisome proliferator-activated receptor-γ coactivator 1α; TFAM: transcription factor A, mitochondria; mtDNA: mitochondrial DNA; SDH: succinate dehydrogenase; DRP1: dynamin-related protein 1; UCP: uncoupling protein; OXPHOS: oxidative phosphorylation; ROS: reactive oxygen species; GFR: global food restriction; ADP: adenosine diphosphate; ATP: adenosine triphosphate; ER: endoplasmic reticulum; MFN/MFN1/MFN2: mitofusin 1/2; PINK1: PTEN induced kinase 1; TG: triglyceride; MFF: mitochondrial fission factor; LC3B: microtubule-associated protein 1A/1B-light chain 3 beta; NRF1: nuclear respiratory factor 1; OPA1: optic atrophy 1; DNM1L: dynamin 1 like; DNM2: dynamin 2; BNIP3: BCL2-interacting protein 3; COX: cytochrome c oxidase; MDH: malate dehydrogenase; GDM: gestational diabetes mellitus; CREB: cAMP-response element binding protein; IDH2: isocitrate dehydrogenase 2; PolG: polymerase γ; SIRT1: sirtuin 1; CPT1: carnitine palmitoyltransferase 1; PDK4: pyruvate dehydrogenase kinase 4; FIS1: mitochondrial fission 1 protein. Mark explanations: upward (↑) and downward (↓) arrows indicate an increase (or upregulation) and a decrease (or downregulation) in the respective processes or levels.

**Figure 3:**
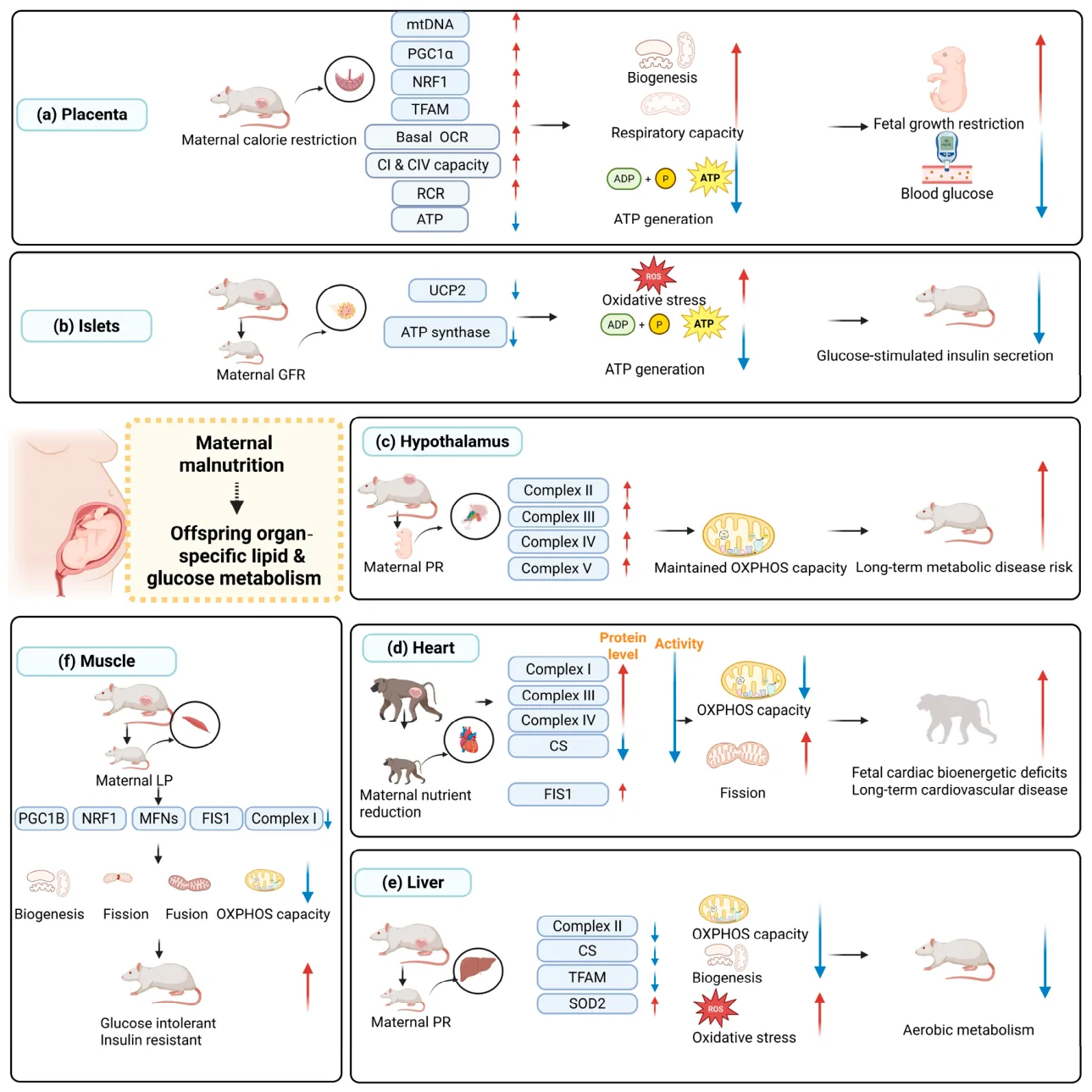
Effects of maternal malnutrition on organ-specific lipid and glucose metabolism disruptions in offspring through mitochondrial dysfunction (Created with BioRender.com. Chuhan Shao. (2026)). (**a**) In the placenta, maternal calorie restriction in rats upregulates mitochondrial biogenesis and respiratory capability but decreases ATP content, leading to fetal growth restriction and reduced blood glucose. (**b**) Maternal GFR in rats decreases UCP2 and ATP synthase, resulting in a lack of glucose-stimulated insulin secretion and ATP content in islets. Additionally, maternal LPD induces oxidative stress in rat islets. (**c**) In fetal offspring of maternal PR, hypothalamic mitochondrial respiratory activity was enhanced. (**d**) Maternal nutrient reduction in baboons triggers increased fission and impaired OXPHOS activity despite increased complex expression. This drives fetal cardiac bioenergetic deficits and cardiovascular disease risk. (**e**) In the liver, maternal LPD in rats reduces mitochondrial biogenesis, density, and complex II, while elevating SOD2, which ultimately decreases aerobic metabolism. (**f**) Maternal LPD downregulates muscle mitochondrial biogenesis, fusion, fission, and respiratory capability in rats, culminating in glucose intolerance and insulin resistance. Mark explanations: upward (↑) and downward (↓) arrows indicate an increase (or upregulation) and a decrease (or downregulation) in the respective processes or levels. CI: complex I; CIV: complex IV; mtDNA: mitochondrial DNA; PGC-1α: peroxisome proliferator-activated receptor-γ coactivator 1α; NRF1: nuclear respiratory factor 1; TFAM: transcription factor A, mitochondria; OCR: oxygen consumption rate; RCR: respiratory control ratio; ATP: adenosine triphosphate; ADP: adenosine diphosphate; GFR: global food restriction; UCP2: uncoupling protein 2; ROS: reactive oxygen species; PR: protein restriction; OXPHOS: oxidative phosphorylation; CS: citrate synthase; FIS1: mitochondrial fission 1 protein; SOD2: superoxide dismutase 2; LP/LPD: low protein/low-protein diet; PGC-1β: peroxisome proliferator-activated receptor-γ coactivator 1β; MFNs: mitofusins (mitofusin 1/2).

Maternal nutritional imbalance leads to the pathophysiology of metabolic disorders and changes in multiple tissues and organs through mitochondrial dysfunction. This altered pregnancy environment can subsequently affect lipid and glucose metabolic status, energetics, and endocrine function. These alterations mediate fetal developmental programming and evoke pronounced metabolic complications, including in the placenta, islet cells, hypothalamus, heart, liver, and muscle (Figure 2 and Figure 3, Table 1).

### 5.1. Placenta

The placenta serves as a unique organ shared by the mother and fetus and is essential for promoting healthy fetal growth and development. Given its intense energy requirements, any mitochondrial impairment in this tissue can result in significant long-term risks for the offspring. Conditions such as maternal obesity or GDM are known to trigger mitochondrial abnormalities like higher levels of ROS and mtDNA damage in the placenta [79].

Clinical data link maternal obesity to higher incidences of SGA infants across both sexes, along with increased blood glucose levels in the progeny. To elucidate the underlying mechanisms, maternal consumption of an HFD in mice notably impairs the mitochondrial capacity for FAO, contributing to enhanced transport of lipids to the fetus, excessive lipid buildup [53,80], and increased adiposity in developing offspring [81]. Specifically, in male mouse offspring, maternal obesity was linked to increased levels of mitochondrial complex II, ATP synthase, and the fission-related protein DRP1, thus helping preserve OXPHOS capacity in damaged mitochondria [53]. However, the concurrent downregulation of mitochondrial uncoupling protein 2 (UCP2) expression is observed, indicating that ROS accumulation and cell death occur [53]. This systemic bioenergetic collapse is mirrored in human cohorts, where placentas from mothers with severe obesity or GDM display heightened ROS production, extensive mtDNA damage, and profoundly suppressed mitochondrial respiratory capacity [79,82,83].

Maternal calorie restriction leads to a compensatory increase in mitochondrial biogenesis and respiratory capacity, but ultimately impairs ATP synthesis in rats [72]. Similar oxidative stress and compensatory upregulations of UCP2 and PPARγ are observed in mouse and ovine models, respectively [84,85]. While these mechanisms may temporarily sustain fetal growth, they establish a highly vulnerable metabolic phenotype predisposed to early-onset syndrome [85].

Within the placenta, mitochondrial function is highly governed by different placenta cells. During placental development, trophoblasts differentiate into cytotrophoblasts (CTBs) and syncytiotrophoblasts (STBs), each exhibiting a distinct mitochondrial phenotype. CTBs are characterized by a heavy mitochondrial fraction and are regarded as metabolically dominant [86]. In contrast, STBs contain a lighter mitochondrial fraction and decreased ATP synthase dimerization, resulting in a comparatively lower inherent capacity for ATP generation via OXPHOS [86]. Cultured trophoblasts from human GDM pregnancies exhibit a profound 50% reduction in mitochondrial respiration [87]. Similarly, human umbilical vein endothelial cells (HUVECs) exposed to adverse metabolic conditions demonstrate reduced ATP-dependent proliferation [86]. Ultimately, whether key metabolic engines like complex V (ATP synthase) are activated or inactivated during maternal overnutrition is not a uniform organ-wide event, but intricately depends on the specific cell type and the stage of pathological stress [86].

### 5.2. Islet Cells

Maternal obesity and malnutrition can lead to increased fasting blood glucose levels and impaired oral glucose tolerance test results across generations in rats [77]. Mitochondria play a central role in pancreatic β-cell glucose sensing, and mitochondrial dysfunction can compromise glucose-stimulated insulin secretion (GSIS). Insulin secretion requires an increase in cytosolic ATP production, which ultimately triggers insulin release via exocytosis. However, in rat models, exposure to either a maternal HFD or global food restriction (GFR) severely blunts this bioenergetic response; upon glucose stimulation, the requisite ATP spike is completely blocked in the islets of the offspring [52].

Intriguingly, this secretory failure is driven by tissue-specific maladaptive molecular responses. For instance, prenatal malnutrition increased the gene expression of the biogenesis factor *Pgc-1α* in the islets of rats subjected to GFR [52]. *Pgc-1α* is considered harmful to β-cells and is associated with insulin secretion defects. Concurrently, the observed downregulation of *Ucp2* in male offspring from the HFD and GFR groups, owing to its role in regulating mROS, may contribute to oxidative stress in islets and reduced GSIS [52]. Increased oxidative stress is also evidenced by the F2 generation of grandmother malnutrition, as the levels of markers of oxidative stress (nuclear and mitochondrial 8-oxo-deoxyguanosine) are significantly elevated [77].

### 5.3. Hypothalamus

The hypothalamus is a crucial brain region that plays a dominant role in energy homeostasis and food consumption, with its development closely dependent on maternal nutrition and metabolic status.

Maternal PR alters cellular metabolism, impairing early neuronal development and, ultimately, hypothalamic function [78]. Changes in mitochondrial structure and metabolism occur throughout the differentiation process [88]. During neural differentiation, energy metabolism shifts from glycolysis to OXPHOS to meet higher demands [88]. In the fetal rat offspring of PR-exposed mothers, hypothalamic mitochondrial respiratory activity was enhanced, as shown by upregulated respiratory chain and metabolic genes, elevated membrane potential, and increased protein levels of complexes II–V, primarily in female fetuses. The mtDNA copy number (mtDNAcn) was unchanged, indicating that the difference in respiratory activity stems from metabolic activity, not mitochondrial quantity. These findings suggest enhanced hypothalamic mitochondrial respiratory activity in the fetuses of mothers with PR; further validation via mitochondrial mass and ROS measurement is needed [78].

In contrast to the enhanced mitochondrial respiratory activity observed under maternal PR, maternal overnutrition elicits opposite and maladaptive alterations in offspring hypothalamic mitochondria. Maternal HFD consumption severely impairs the respiratory chain, evidenced by decreased expression of mitochondrial complexes III and V [51]. The resulting accumulation of damaged mitochondria triggers an upregulation of mitophagy, indicated by elevated PINK1 and Parkin levels in offspring, making them more likely to be targeted for autophagosomal degradation in rats [51]. Maternal high-calorie diet/HFD exposure induces hyperinsulinemia and hyperleptinemia in rat offspring, causing impaired glucose sensing, leptin/insulin resistance, and excessive fat accumulation [50]. Paradoxically, rather than restoring metabolic homeostasis, this developmental overnutrition triggers aberrant mitochondrial remodeling characterized by excessive fusion. Specifically, expression of the fusion mediator protein MFN2 is upregulated, whereas that of the fission mediator DRP1 is downregulated in the offspring hypothalamus [50]. This shift toward increased fusion is maladaptive, although reduced fusion with upregulated fission is more commonly observed in energy failure and mitochondrial dysfunction in metabolic disorders. This loss or reduction in DRP1 expression impairs ROS-dependent glucose sensing in hypothalamic neurons and further exacerbates mitochondrial dysfunction [89]. Thus, high-calorie maternal programming drives enhanced mitochondrial fusion and mitophagy and reduced OXPHOS complexes in the offspring hypothalamus as a deleterious response that impairs metabolic sensing and increases lifelong obesity susceptibility [50].

Maternal nutrition perturbs the hypothalamic network with profound cellular specificity. The central melanocortin system relies on a delicate balance between orexigenic neuropeptide Y/agouti-related peptide (NPY/AgRP) neurons and anorexigenic pro-opiomelanocortin (POMC) neurons, and their mitochondria possess completely divergent metabolic requirements and stress responses. For instance, neuron-specific modulation of MFN2 expression in obesity mice models has revealed that MFN2 overexpression in POMC neurons promotes obesity, whereas its deletion in AgRP neurons prevents weight gain [90,91]. Meanwhile, studies in rat models of maternal obesity have shown that offspring exposed to a maternal HFD exhibit increased numbers of orexigenic peptide-expressing neurons in the hypothalamus [51]. A maternal HFD during lactation also increased the ratio of NPY to POMC neurons in mouse offspring [92]. Ultimately, it is this cell-specific mitochondrial remodeling that permanently rewires the hypothalamic architecture, predisposing the offspring to lifelong metabolic disorder susceptibility.

### 5.4. Heart

Unlike tissues with high turnover rates, which can replenish damaged mitochondria through cell division, cardiomyocytes preserve mitochondrial function primarily through fission, fusion, and selective mitophagy to eliminate damaged organelles [54]. When these processes are impaired, mitochondrial fragmentation, defective mitophagy, and cell death occur, eventually leading to heart failure [93]. This is a major pathogenic mechanism underlying diabetic cardiomyopathy in adults [93]. Perinatal exposure to maternal overnutrition and diabetes causes cardiac dysfunction in neonates and has long-term effects on offspring hearts, such as abnormal mitochondrial morphology [55,56], increased oxidative stress, impaired mitochondrial dynamics [41], reduced respiratory capacity and FAO [55,56,57], and, ultimately, myocardial lipid accumulation [54], increased heart weight [55], and impaired systolic and diastolic function [56].

Offspring exposed to perinatal high-nutrition conditions exhibit numerous short, wide, and fragmented mitochondria in cardiomyocytes, with markedly reduced fission and fusion activity, particularly impaired fusion, as observed in rat models [55,56]. This effect is even more pronounced in offspring exposed to both maternal diabetes and an HFD, in whom almost no fission or fusion events can be observed, indicating severe mitochondrial damage and loss of dynamics, a state that may herald apoptosis [55,56]. Under conditions of excessive maternal circulating fuels, downregulation of MFN1 expression significantly reduces mitochondrial fusion in offspring cardiomyocytes, whereas MFN2 protein levels are slightly increased. In the heart, MFN2 predominantly sensitizes cardiomyocytes to mitochondrial permeability transition and cell death under stress [56]. This abnormal fission and decreased fusion lead to the formation of mitochondrial fragments containing damaged mtDNA and exhibiting impaired OXPHOS [41].

In rats, normal hearts at 6 months of age rely primarily on OXPHOS and FAO for ATP production [57]. To meet this energy demand, offspring exposed to maternal high-nutrition conditions have increased mitochondrial numbers and mtDNAcn; however, this does not meet energy needs. Their FAO and respiratory capacity decreased [55,56,57]. This decline is accompanied by reduced expression of mitochondrial respiratory complex components such as ATP synthase F1 subunit beta (ATP5B) [29], which likely contributes to myocardial lipid accumulation in rats [54]. Crucially, this metabolic collapse is conserved in sheep [58].

Mitochondrial function is a critical modulator of cell death in models of cardiac injury. In rodent models, maternal exposure to an HFD induces cardiac mitochondrial damage and decreases mitophagy [54]. Defective mitophagy results in the accumulation of dysfunctional mitochondria, which may subsequently induce cardiomyocyte death [41]. Cardiomyocytes from 12-month-old rat male offspring exposed to maternal diabetes exhibit faster loss of MMP and accelerated mitochondria-mediated cell death under stress, suggesting that under increased energy demand, this could translate into more severe cardiac injury [57].

Furthermore, the profound impact of maternal nutrient reduction (MNR) on fetal cardiac mitochondria has been elegantly demonstrated in highly translational baboon models [73,74]. During gestation, MNR induces a paradox within the fetal heart. Specifically, nutrient restriction significantly diminishes overall mitochondrial density, as evidenced by decreased citrate synthase levels, and heavily disrupts mitochondrial architecture, leading to sparse, disarranged cristae [73,74]. In a desperate biological attempt to rescue energy production, the fetal heart mounts a compensatory response characterized by an increased mtDNAcn and the upregulation of specific OXPHOS complex subunits (including complexes I, III, and IV) [73,74]. However, this molecular compensation is functionally futile. Despite the higher abundance of OXPHOS proteins, the enzymatic activities of complexes I, II, and III are significantly impaired [73].

Within cardiomyocytes, mitochondria exist as highly heterogeneous populations that perform different physiological roles. Based on their spatial organization, cardiac mitochondria are compartmentalized into distinct subpopulations, including intermyofibrillar, subsarcolemmal, and perinuclear locations [94]. However, despite this well-established subcellular complexity, current investigations into the transgenerational effects of maternal metabolic stress on cardiac mitochondria have largely relied on whole-tissue analyses.

### 5.5. Liver

MAFLD has emerged as a revised term for fatty liver disease and is distinct from nonalcoholic fatty liver disease (NAFLD). MAFLD is diagnosed based on the presence of fatty liver along with overweight or obesity, type 2 diabetes mellitus, or lean or normal weight with evidence of metabolic dysregulation [95]. The current prevalence of MAFLD, according to a large pooled analysis, is estimated to be 39% worldwide [96]. A threefold higher risk of developing MAFLD was observed in the offspring of obese mothers than in those of normal-weight mothers in human clinical cohorts [97]. As a reflection of its high energy metabolic demand, the liver is among the most mitochondria-rich organs in the body [98]. This high metabolic reliance raises the possibility that maternal diet mediates the intergenerational effect on offspring MAFLD susceptibility through lasting alterations in hepatic mitochondrial physiology.

According to the prevailing view, in response to high hepatic fatty acid loads, mitochondria initially increase OXPHOS capacity, a compensatory adjustment that increases mitochondrial ROS production.

In genetic models of maternal insulin resistance, complex IV subunit 1 is upregulated in mice [68], which is consistent with the increase in adaptive mitochondrial capacity in early-stage MAFLD [42]. Excessive maternal fructose consumption increases serum triglyceride (TG) concentrations without inducing hepatic lipid deposition. However, enhanced OXPHOS was observed in the offspring of mothers fed a fructose-supplemented diet, as evidenced by increased activities of mitochondrial complexes II and IV and elevated hepatic voltage-dependent anion channel 1 (VDAC1) expression at weaning in guinea pig offspring [67]. VDAC1 is a critical regulator of mitochondrial metabolic function, facilitating efficient energy transfer and ETC activity. Its downregulation has been shown to impair ETC function and ATP production [67]. The simultaneous increases in VDAC1 expression and ETC efficiency are likely driven by the heightened metabolic demands of neonatal development. Crucially, this transient OXPHOS upregulation represents an adaptive response to the increasing hepatic lipid load, temporarily protecting the neonatal liver from overt lipotoxicity and steatosis during the early phases of MAFLD pathogenesis [99].

However, this upregulated OXPHOS is subsequently downregulated as the disease progresses. In a mouse model of maternal obesity-induced liver steatosis in 12-month-old offspring, significant decreases were observed in the protein expression of mitochondrial respiratory chain complexes I, III, IV, and V [69]. Similarly, multigenerational Western diet (WD) feeding downregulated genes encoding mitochondrial OXPHOS subunits, leading to reduced abundance and activity of complexes III, IV, and V, which was further exacerbated with the progression of fibrosis [68]. Additionally, the hepatic mtDNA copy number in offspring of WD-fed mothers increased significantly prior to fibrosis, but decreased once fibrosis developed [68]. This suggests a compensatory response to increased free fatty acids flux before the development of fibrosis [99]. In contrast, this adaptive mechanism is attenuated in mice with fibrosis [99].

Beyond respiratory dysfunction, reduced dynamics, an unbalanced fission/fusion cycle, and mitophagy arrest are observed across the MAFLD spectrum [33]. Concurrently, the biogenesis machinery becomes uncoordinated; while *Nrf1* may spike aberrantly during fibrosis, the master regulator *Pgc-1α* is downregulated, leading to diminished mitochondrial content and citrate synthase activity [68].

With respect to mitochondrial dynamics, only the protein content of the fusion marker OPA1 was upregulated by maternal GDM, indicating that the inner mitochondrial membrane containing the ETC is more sensitive to alterations in maternal nutrition [100]. Similarly, Opa1 expression was increased in mice subjected to multigenerational WD feeding but decreased upon fibrosis development. Conversely, the expression of the fission markers *Drp1* and Dynamin 2 (*Dnm2*) is increased in fibrotic mice [68]. Enhanced fusion was detected, especially on the inner membrane, but the degree of fusion decreased as fibrosis progressed. The degree of mitochondrial fission was increased, which is coincident with that in MAFLD pathogenesis [101]. Furthermore, maternal Western diet exposure suppresses *Bnip3* expression, effectively arresting mitophagy and preventing the clearance of damaged organelles in mice [68]. Cells from offspring of obese dams are more susceptible to apoptotic stimuli [69]. This maternal overnutrition leads to increased ROS production, which impedes the fission/fusion cycle, arrests mitophagy, and leaves hepatocytes with damaged mitochondria, ultimately inducing apoptosis [102].

Beyond nutrient excess, maternal undernutrition also inflicts comprehensive damage on the hepatic mitochondrial network. In Wistar rat models, exposure to a maternal LPD represses mitochondrial biogenesis by downregulating TFAM, translating into a profound decrease in overall mitochondrial density, as evidenced by diminished citrate synthase levels. Furthermore, this structural depletion is accompanied by a suppression of OXPHOS complex II. This compromised bioenergetic state inevitably triggers elevated oxidative stress. Although the offspring’s liver attempts to mount a compensatory antioxidant defense by significantly upregulating superoxide dismutases (SOD1 and SOD2), this protective response is insufficient to rescue the energetic deficit [75].

While the aforementioned molecular alterations provide insight into global hepatic dysfunction, evaluating the liver as a homogeneous tissue obscures the mitochondrial characteristics and metabolic roles displayed in different spatial zonation. In the periportal zone (Zone 1), located near the portal vein, hepatocytes typically harbor enlarged, spherical mitochondria. This specific structural adaptation facilitates efficient oxygen and nutrient transfer. As a result, these periportal mitochondria are efficient at generating ATP via OXPHOS and fatty acid oxidation. In contrast, at a distance of approximately 300 μm in the pericentral zone (Zone 3), mitochondria transition into an elongated, tubular network. This shape favors matrix-driven enzymatic processes, including citrate synthesis and pyruvate oxidation, which are vital for lipogenesis [103].

Crucially, this intrinsic metabolic zonation dictates the pathological response to perinatal metabolic stress. A prime clinical illustration of this is pediatric metabolic dysfunction-associated steatotic liver disease (MASLD). In adults, MASLD conventionally manifests with centrilobular (Zone 3) damage. Conversely, pediatric MASLD, a condition where disease vulnerability and severity are strongly driven by detrimental perinatal exposures, is characterized primarily by periportal (Zone 1) fibrosis, inflammation, and steatosis. This distinct histological divergence underscores the unique vulnerability of the highly oxidative, OXPHOS-reliant mitochondria within Zone 1 hepatocytes to disruptions caused by an unfavorable maternal metabolic environment during fetal development [104]. Further direct evidence is needed to detect these transgenerational effects in different zones.

### 5.6. Muscle

Skeletal muscle accounts for 40–50% of total body mass and serves as a central player in the pathogenesis of obesity and insulin resistance. Postnatally, the number of muscle fibers remains fixed. Consequently, any disruption in fetal skeletal muscle development can lead to lifelong decreases in muscle mass and performance, thereby increasing the possibility of developing diabetes later in life [105]. Muscle lipid accumulation has been demonstrated in the soleus muscles of rat offspring of mothers fed an HFD [106]. Intrauterine exposure to hyperglycemia or excessive nutrient supply has been repeatedly linked to mitochondrial dysfunction in offspring skeletal muscle, characterized by aberrant mitochondrial morphology, diminished respiratory capacity, suppressed biogenesis, and disrupted dynamics, all of which impair progeny glucose homeostasis.

Maternal HFD consumption alters mitochondrial ultrastructure, resulting in the loss of organelles with electron-lucent matrix and disrupted or absent cristae. In the offspring of HFD-fed or streptozotocin-treated diabetic dams, the expression of PGC-1α and NRF1 is markedly reduced in pigs [59]. Similarly, in mice, these conditions are accompanied by lower ATP levels and decreased mtDNA copy number, reflecting compromised mitochondrial biogenesis [60]. This biogenesis defect appears to be associated with fetal hyperglycemia, which suppresses cAMP-response element binding protein (CREB) phosphorylation and consequently downregulates *Pgc-1α* transcription [60]. Defects in offspring mitochondrial biogenesis related to maternal obesity are related to the suppression of isocitrate dehydrogenase 2 (IDH2) expression via H3K9me3 histone modification at the IDH2 promoter in mice [65], which contributes to systemic insulin resistance. The presence of mitochondrial polymerase γ (PolG) mutation exacerbates adverse changes in offspring muscle under maternal HFD conditions in mice [64]. Mitochondrial dynamics are also impaired, as indicated by decreased mRNA expression of *Mfn1* and *Mfn2*, reflecting defective fusion.

Respiratory function is likewise diminished in baboons [63], although sometimes in the offspring soleus of HFD-fed mothers, increased activity of the respiratory chain can be observed as a compensatory mechanism in the presence of mitochondrial injury [106]. The expression of genes involved in oxidative metabolism and the ETC is significantly decreased in the soleus muscle of offspring from GDM rat models [61]. In another study, the expression of UCP3, the most abundant uncoupling protein in skeletal muscle, decreased, promoting the accumulation of ROS [106]. Moreover, the activities of succinate dehydrogenase (SDH), a pivotal respiratory chain enzyme [50], and malate dehydrogenase (MDH), which supports respiratory capacity [51], decreased in the HFD group. This provides further evidence that a maternal HFD or mixed diet compromised mitochondrial function in offspring skeletal muscle in pigs [59]. Changes in mitochondria-associated proteins are essential for maintaining skeletal muscle oxidative performance. In fetal offspring muscle, cytochrome c levels decrease, while the catalytic subunits of cytochrome c oxidase are affected, together with reduced mtDNA content and lower citrate synthase activity in baboons [63]. FAO capacity is impaired, as evidenced by the decreased activity of β-hydroxyacyl-CoA dehydrogenase [63]. Crucially, this mitochondrial vulnerability is strictly conserved in a robust Japanese macaque model exposed to a Western-style diet for nine years prior to pregnancy. Fetal skeletal muscle demonstrates an attempt to rescue energy homeostasis. This chronic primate model exhibits significant upregulation of regulatory sirtuins (SIRT3) and UCP2 and UCP3 to manage severe lipotoxic stress, alongside increased specific activities of OXPHOS complexes I and IV. However, this molecular overactivation fails to prevent structural collapse and respiratory capacity decline [66].

Fetal tissue growth and metabolism are strongly dependent on maternal nutrient supply. A maternal low-protein diet (LPD) adversely affects various organs, often sparing the brain at the expense of others. Skeletal muscle is particularly vulnerable to PR [107]. Similarly, gestational protein deficiency results in fewer muscle fibers; impaired mitochondrial function; and reduced biogenesis, fusion, and fission in rodent offspring [16,76]. When mitochondrial dynamics are perturbed, skeletal muscle mitochondria display lower respiratory efficiency and bioenergetic capacity, limiting their ability to satisfy increased energy requirements in rats [76]. PR also disrupts complex I integrity and dynamics while altering substrate oxidation gene expression, suggesting a shift toward preferential utilization of fatty acids over pyruvate. This inefficiency in the metabolism of both glucose and lipids may increase circulating glucose levels and underlie the glucose intolerance observed in these models [108].

Mammalian skeletal muscle is broadly categorized into three predominant fiber types: slow-twitch oxidative (Type I), fast-twitch oxidative (Type IIA), and fast-twitch glycolytic (Type IIB or IIX) fibers. Oxidative fibers are distinguished by a rich capillary supply and abundant mitochondria, depending primarily on OXPHOS to synthesize ATP. Conversely, glycolytic fibers exhibit restricted mitochondrial volume, coupled with a denser concentration of actin and myosin contractile proteins [109]. Crucially, exposure to a maternal HFD exerts profoundly detrimental effects on the structural integrity of the offspring’s skeletal muscle by driving a pathological fiber-type transition. Specifically, pre-pregnancy and maternal HFD exposure triggers a progressive shift that depletes mitochondria-rich, highly oxidative populations, resulting in a marked reduction in both Type I and Type IIA fibers. Concurrently, this metabolic stress promotes a transition to anaerobic Type IIB fibers with low mitochondrial density [59,64,65]. In the mouse models, a protein-restricted diet during lactation poses a loss of type-IIA muscle fibers and reduced muscle fiber size as well [110].

Collectively, maternal nutritional stress elicits profound, yet highly tissue-specific, mitochondrial maladaptations across offspring organs. While the specific manifestations differ, ranging from impaired glucose-stimulated insulin secretion in pancreatic islets to defective fatty acid oxidation and altered fission/fusion dynamics in the liver, heart, and skeletal muscle, these organ-specific mitochondrial dysfunctions consistently act as the central mechanistic hubs linking the adverse maternal environment to systemic metabolic disease.

## 6. Therapeutic Targets to Rescue Mitochondrial Function in Later Generations

### 6.1. Dietary Interventions

Pregnancy and lactation are the most sensitive and effective windows for maternal dietary/lifestyle interventions, conferring lifelong benefits to both mothers and offspring.

A Mediterranean diet (MedDiet) is distinguished by high consumption of fruits, vegetables, whole grains, nuts, and legumes, moderate consumption of poultry and fish, and limited red meat, with olive oil as the main fat source [111]. The diet is abundant in unsaturated fats and most of its protein and fat are from plant sources. This replacement of saturated fats with unsaturated fats improves cardiometabolic outcomes, maybe by enhancing endothelial function, reducing inflammatory eicosanoids, and targeting lipid and glucose metabolism to improve cardiometabolic outcomes [112]. MedDiet reduces mROS production and ameliorates mitochondrial damage and apoptosis [113]. Adherence to the MedDiet during pregnancy not only improves maternal outcomes and acts as a protective factor against the development of GDM but also confers long-term benefits to offspring, including improved blood pressure, reduced cardiometabolic risk, and lower adiposity in humans according to a systematic review [114].

The offspring of mothers who switched from an HFD to a standardized chow diet before weaning or during lactation presented a significantly lower risk of obesity than the rat offspring of mothers maintained on an HFD did [13]. However, excessive caloric restriction during pregnancy can lead to fetal IUGR in mice [13] and adversely affect mitochondrial function across various offspring organs. Therefore, improving dietary quality (e.g., nutrient composition and food diversity), rather than merely restricting dietary quantity (caloric restriction), appears to be a more promising and superior intervention strategy. Postnatal dietary intervention in offspring is also effective. In mice, when offspring of HFD-fed dams are switched to a standard chow diet after weaning, mitochondrial FAO is increased, leading to normalization of glucose tolerance and lipid profiles, thereby reversing the adverse metabolic programming induced by maternal exposure to an HFD [115].

Given that excessive mitochondrial ROS (mROS) and oxidative stress are central drivers of transgenerational metabolic dysfunction, targeted antioxidant therapy might be a rational intervention. Supplementation with natural compounds during pregnancy is another powerful approach to counteract the detrimental effects of maternal nutrient excess on offspring mitochondrial and metabolic health. Antioxidants can upregulate mitochondrial biogenesis and restore impaired mitochondrial function [116].

Specific compounds have demonstrated protective effects in various models. Resveratrol, a natural polyphenol antioxidant, promotes browning and thermogenesis of white adipose tissue in the mouse offspring of HFD-fed mothers when supplemented during pregnancy, preventing obesity in male offspring [117]. Moreover, maternal melatonin intake during gestation ameliorates hepatic steatosis in pups of mothers exposed to both an HFD and microplastics [118]. Similarly, maternal consumption of L-malic acid in pigs improves antioxidant capacity and glucose metabolism in piglets’ offspring [119]. The supplementation of antioxidant vitamins in HFD-fed rat dams reduces offspring obesity and restores normal glucose tolerance [13]. Furthermore, a diet abundant in antioxidants (Vitamin E, coenzyme Q10 and α-lipoic acid) prevented β-cell loss and apoptosis and promoted the formation of dual hormone-expressing endocrine cells in nicotine-exposed male offspring [120].

In summary, optimizing maternal dietary quality and timely postnatal dietary correction and antioxidant supplementation during critical developmental windows represent safe, effective, and translatable strategies to overcome the intergenerational cycle of obesity and metabolic disease transmitted via mitochondrial dysfunction.

### 6.2. Exercise

Pregnancy is often accompanied by a decrease in physical activity. Moderate exercise during pregnancy helps reduce the risk of macrosomia and adiposity in offspring and enhances their exercise capacity and cardiac autonomic health in humans [121], with these effects potentially mediated through improvements in mitochondrial function. In mothers with GDM, exercise during pregnancy mitigates early-life weight gain and hepatic fat accumulation induced by a maternal HFD, enhances offspring liver mitochondrial respiration capacity in rats [122], and restores GDM-impaired mitochondrial biogenesis in a rat model [70,123,124]. A maternal HFD leads to excessive activation of ryanodine receptor 2 (RyR2) oxidation induced by mitochondrial ROS, and maternal exercise alleviates this effect while enhancing offspring mitochondrial respiration, thereby attenuating the transgenerational susceptibility to cardiovascular diseases caused by an HFD in guinea pigs [53]. Similarly, maternal exercise can counteract the reduction in citrate synthase activity and elevated ROS levels in cardiac oxidative metabolism in rat offspring due to PR during lactation and pregnancy [125].

Clinically, the principles of exercise prescription for pregnant women do not differ from those for the general population. The goal is to gradually achieve at least 20–30 min of moderate-intensity exercise per day on most or all days of the week. For obese pregnant women, starting with low-intensity, short-duration exercise and gradually increasing the duration or intensity of exercise as tolerated is recommended. For most pregnant women, walking and water-based exercises are safe and effective options [126].

### 6.3. Pharmacological Interventions

Metformin reduces body weight in type 2 diabetes patients, and its efficacy in limiting GWG in obese pregnant women has also been observed in human clinical trials [127]. However, the impact of prenatal metformin exposure on metabolic outcomes in adult offspring remains debated.

Maternal metformin intervention during obese pregnancy was shown to cause excessive adiposity and white adipose tissue inflammation in male offspring in mice [128]. In contrast, other studies have shown that administering metformin before and during pregnancy under maternal HFD conditions reduces fat accumulation and improves glucose tolerance in mouse offspring, demonstrating a protective effect [129].

NAD^+^ participates in oxidation‒reduction reactions, and nicotinamide mononucleotide (NMN) serves as a precursor of NAD^+^, enhancing NAD^+^ biosynthesis. Partial restoration of oocyte quality in HFD-fed mice following NMN administration was associated with recovery of mitochondrial function and actin dynamics, as well as reductions in meiotic defects, DNA damage, ROS accumulation, and aberrant lipid droplet distribution within oocytes [130]. Nevertheless, the clinical anti-obesity effects of these agents, especially during pregnancy, still require thorough evaluation of safety and efficacy.

Postnatal drug intervention in offspring exposed to maternal overnutrition was also investigated. Fibroblast growth factor 21 (FGF21) agonism improves MASH by enhancing mitochondrial function and reducing oxidative stress in mice [131]. For instance, FGF21 supplementation is effective for upregulating mitochondrial biogenesis in FGF21-supplemented offspring exposed to a maternal WD [101]. Oral administration of simvastatin, an HMG-CoA reductase inhibitor, or metformin to young HFD offspring reversed maternal HFD-induced changes in mitochondrial biogenesis and alleviated ROS production, thus decreasing hypertension in rats [132]. NMN administration in the mouse offspring of obese mothers resulted in a modest increase in glucose tolerance and improved the expression of mitochondrial functional markers [133,134].

### 6.4. Mitochondrial Transplantation

In recent years, mitochondrial transplantation has attracted considerable interest as a strategy for functional tissue repair. This technique involves artificially supplementing or replacing defective mitochondrial networks with respiration-competent, viable mitochondria. The core methodology includes isolating mitochondrial preparations from healthy donor tissues and delivering them, via local or circulatory injection, to regions adjacent to dysfunctional mitochondrial sites [28]. For offspring with metabolic disturbances resulting from nutritional imbalances, mitochondrial transplantation may offer a promising therapeutic avenue [135].

Impaired mitochondrial adaptation, notably deficiencies in respiratory and metabolic capacity, is a central driver of the progression of insulin resistance and MAFLD. Following mitochondrial transplantation, lipid accumulation gradually decreases, accompanied by reduced serum transaminase activity. Studies have further demonstrated that transplantation significantly decreases ROS and malondialdehyde levels while increasing glutathione (GSH) content and superoxide dismutase (SOD) activity in mice [136]. These findings suggest that mitochondrial transplantation can enhance lipid metabolism and mitigate oxidative hepatocyte damage induced by an HFD.

Similarly, in an HFD-induced MAFLD model, fat deposition in steatotic hepatocytes was decreased following mitochondrial transfer from bone marrow-derived mesenchymal stem cells (BMSCs). In obese mice, recipient cells exhibited elevated OXPHOS activity, increased ATP production, restored MMP, and decreased ROS levels, alongside improved liver function, decreased steatosis, attenuated weight gain, and improved systemic glucose and lipid metabolism [137]. Furthermore, human adipose-derived mesenchymal stem cells (MSCs) have been shown to mediate mitochondrial transfer to pancreatic islets, thereby increasing insulin secretion [138].

Despite its compelling therapeutic potential, mitochondrial transplantation poses significant translational risks that must be carefully evaluated.

One concern is mitochondrial heteroplasmy. Divergent nonpathologic mtDNA heteroplasmy (DNPH) is defined as the presence of >1 mtDNA variant in the same cytoplasm, which can result from new medical technologies like mitochondrial replacement and transplantation. DNPH may cause incompatibilities between donor and recipient mtDNAs, metabolic stress, cell death, and ultimately pathogenic and degenerative diseases [139].

Another concern is that the impact of this technology may lead to nuclear remodelling. Donor mitochondria may influence the interaction between mitochondria and the nucleus, resulting in consequential effects on the nuclear epigenome and transcriptome [140]. Evidence from cybrid cell models has demonstrated that such mito-nuclear incompatibility can lead to metabolic disarray [141].

Beyond mito-nuclear mismatch, another concern is post-transplantation immune responses. The mitochondria commonly applied for transplantation are usually autologous transplantation; however, for patients with systemic mitochondrial defects, only allogeneic transplantation is applicable. Allogeneic transplantation may provoke inflammatory cascades that are seemingly linked to the allograft dysfunction derived from circulating mitochondrial DAMPs (damage-associated molecular patterns) [142]. Although clinical evidence supporting mitochondrial transplantation for metabolic diseases remains limited, it represents an emerging and potentially impactful direction for future targeted therapies.

**Figure 4:**
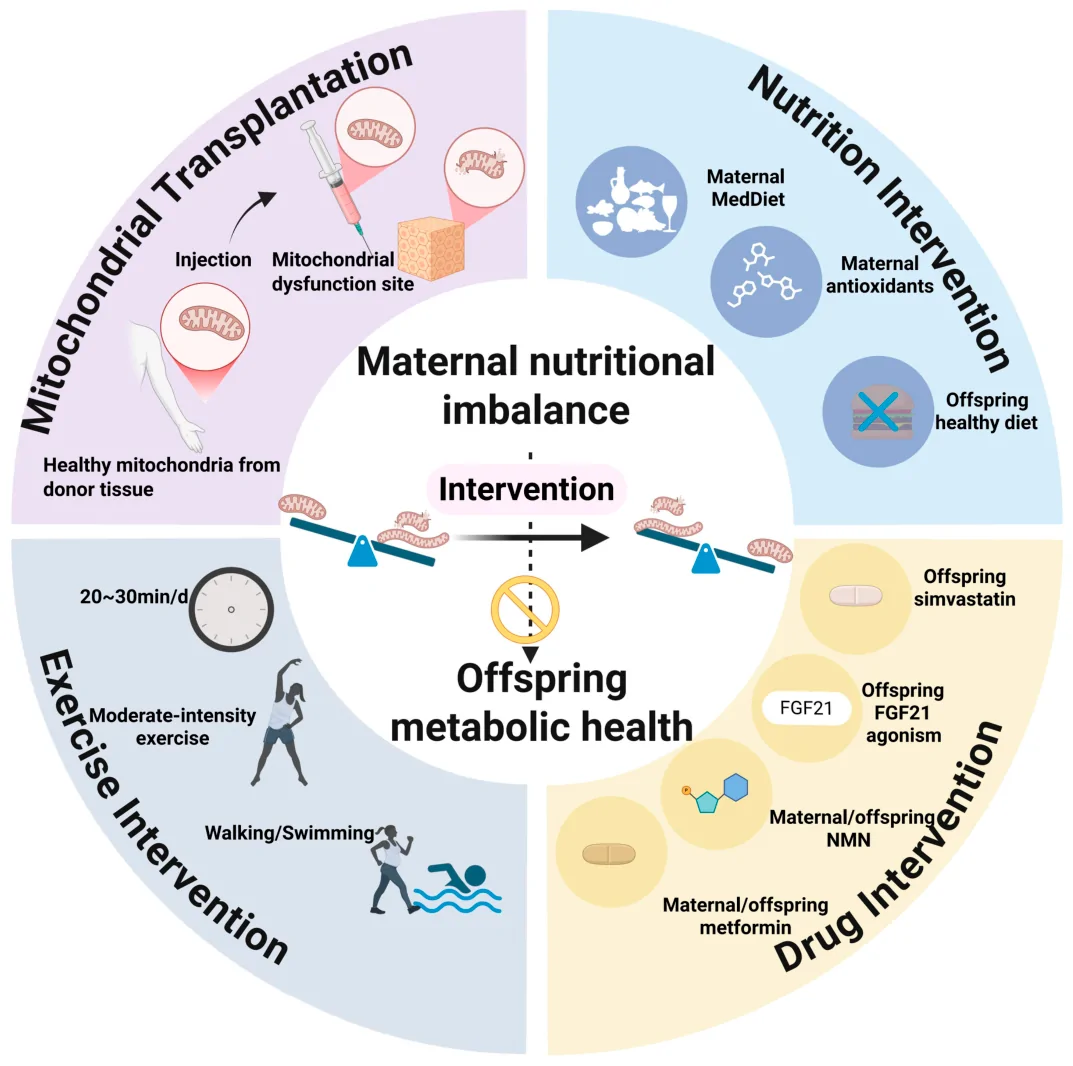
Strategies for existing interventions to improve intergenerational metabolic disease transmission through rescuing mitochondrial dysfunction (Created with BioRender.com. Chuhan Shao. (2026)). Nutrition intervention includes a change in maternal dietary pattern to the Mediterranean diet, supplementation of maternal antioxidants, and persistence on a healthy diet in offspring. Drug interventions including the mother/offspring taking metformin and NMN, or the offspring taking FGF21 or simvastatin, are promising. Maternal exercise, such as walking and swimming, rescues harm due to obesity. Mitochondrial transplantation to mitochondrial dysfunction sites is a promising future direction towards mitochondrial dysfunction due to maternal nutritional imbalance. MedDiet: mediterranean diet; NMN: Nicotinamide Mononucleotide; FGF21: fibroblast growth factor 21.

In summary, targeting mitochondrial dysfunction presents a highly promising therapeutic frontier for breaking the cycle of inherited metabolic disorders. While foundational lifestyle modifications offer safe and effective mitigation, emerging pharmacological interventions and cutting-edge techniques like mitochondrial transplantation hold immense translational potential for directly rescuing transgenerational mitochondrial defects (Figure 4).

## 7. Discussion, Challenges and Future Perspectives

While the profound impact of maternal nutrition on offspring mitochondrial health is clearly established, a critical mechanistic debate remains regarding whether mitochondrial dysfunction acts as a primary programmed event or a secondary consequence of other metabolic stress. On one hand, evidence supports a direct programming effect. For example, a maternal HFD induces persistent epigenetic modifications, like the hypermethylation of the *Pgc-1α* promoter, which is detectable in offspring skeletal muscle at birth and directly drives age-dependent metabolic dysfunction in mice [143]. On the other hand, substantial literature argues that mitochondrial impairment is a secondary response to programmed metabolic stressors, such as lipotoxicity and endoplasmic reticulum (ER) stress. For instance, treating obese female mice with an ER stress inhibitor restores TFAM and DRP1 levels, as well as mtDNA content in oocytes, which may indicate that obesity before conception results in ER stress-driven mitochondrial loss in offspring [144]. Furthermore, ROS-induced lipotoxicity compromises mitochondrial–ER interactions and triggers an ER stress response. This results in a significant leakage of calcium from the ER, which in turn drives excessive calcium influx into the mitochondria and ultimately disrupts their function [27]. Thus, mitochondrial dysfunction represents a synergistic result of direct or indirect influence by maternal nutrition.

Another critical consideration is the sex-specific nature of mitochondrial responses in male and female offspring across different tissues. In the absence of transgenerational programming, although the direction and magnitude of these disparities heavily depend on tissue type, age, hormonal status, and the metabolic microenvironment, consistent patterns have emerged [145]. In multiple experimental models, female tissues typically exhibit a fusion tendency of the mitochondrial network, enhanced mitochondrial quality control, a greater antioxidant buffering capacity, and greater reliance on fatty acid oxidation during endurance exercise and metabolic stress [146,147,148]. Conversely, male tissues often display an increased susceptibility to mitochondrial fragmentation, impaired mitochondrial adaptation with aging, and greater overall oxidative vulnerability [149,150]. Although current studies on offspring mitochondria align with those in non-transgenerational settings, the influence of sex differences on mitochondrial function and its consequences in developmental programming remains a large gap to be further investigated. These could originate very early, as sex defines the cellular responses of murine embryonic cells to stressors and hormones in vitro [151]. Those sex differences may be attributed to sex hormones’ effects on mitochondrial biogenesis, OXPHOS, ROS production, and quality control [145].

Although the primary focus of this review is the maternal environment, it is critical to acknowledge the emerging evidence showing that paternal metabolic health during the preconception period affects offspring metabolism. In humans, paternal obesity is associated with decreased mitochondrial respiratory capacity in infant mesenchymal stem cells, independent of maternal intrauterine factors [152]. Mechanistically, this paternal transmission is driven by nutrition-sensitive, sperm-borne epigenetic factors. For instance, paternal high-fat diets upregulate sperm-borne mitochondrial tRNAs and their fragments, which are transferred to the oocyte at fertilization to govern early-embryo transcription and affect offspring metabolic health in mice [153]. Similarly, paternal obesity induces specific microRNAs (such as let-7d/e) in sperm, which impair oxidative metabolism and mitochondrial activity in the adipose tissue of mouse offspring [154]. Crucially, transgenerational metabolic health is shaped by the interplay of both parental environments. Remarkably, maternal exercise before and during gestation has been shown to attenuate the mitochondrial dysfunction, skeletal muscle insulin resistance, and impaired insulin secretion triggered by paternal obesity in rat models [155]. Taken together, these findings suggest that while the maternal lineage directly provides the mitochondrial genome and the immediate intrauterine environment, maternal–paternal interaction is vital to transgenerational mitochondrial programming.

The strength of this review lies in its comprehensive synthesis of organ-specific mitochondrial dysfunction, providing a detailed mechanistic link between maternal nutritional status and the transgenerational transmission of metabolic damage. However, the present review study has several methodological limitations. As a narrative review rather than a systematic review, it lacks a quantitative meta-analysis, and our literature search was restricted to English-language publications which may inherently introduce language biases. Furthermore, regarding current research gaps in the wider field, there remains a heavy reliance on whole-tissue analyses that obscure spatial cellular heterogeneity, a scarcity of human-based clinical evidence, and a lack of early fetal biomarkers to rapidly identify mitochondrial dysfunction driven by the early nutritional environment. Current research challenges mainly focus on two critical frontiers. First, the field’s heavy reliance on whole-tissue analyses severely obscures the profound cellular heterogeneity and spatial zonation of mitochondrial adaptations, requiring an urgent shift toward single-cell and spatial multi-omics approaches. Moreover, while mitochondrial transplantation offers a promising therapeutic avenue, its clinical translation faces significant technical hurdles.

Future studies addressing mitochondrial dysfunction-mediated metabolic disorder transmission across generations are needed. First, investigating how the interplay among mitochondrial fission, fusion, mitophagy, biogenesis, and energy metabolism contributes to the pathogenesis of maternally transmitted disruptions in lipid and glucose homeostasis induced by nutritional imbalance is essential. Second, advances focused on humans are still lacking; clinical evidence is needed to determine whether mitochondrial dysfunction plays a vital role in metabolic disruption transmission. Additionally, biomarkers that can quickly identify mitochondrial dysfunction in the fetus during pregnancy are needed for immediate intervention. Finally, while mitochondrial transplantation is advancing in mature organs and is utilized in oocytes to prevent inherited mtDNA diseases, its potential application as a therapeutic strategy for maternal overnutrition-induced mitochondrial dysfunction remains an exciting and unexplored frontier.

In summary, transgenerational mitochondrial programming is a complex interplay of epigenetic, metabolic, sex-specific, and paternal factors. Future breakthroughs rely on utilizing spatial multi-omics, identifying early clinical biomarkers, and resolving the translational hurdles of mitochondrial therapies.

## 8. Conclusions

Mitochondrial aberrations can be inherited intergenerationally and present as disturbances in energy metabolism and compromised quality control mechanisms within offspring mitochondria. Their performance in offspring organs after exposure to maternal nutritional imbalance may change because of organ-specific oxygen demand, different cell differentiation capacities in various tissues, and different adaptive responses of mitochondria. These responses may further evolve during disease progression toward either adaptive or maladaptive outcomes.

Current therapeutic strategies predominantly focus on dietary adjustments, supplementation with antioxidants, pharmacological modulation, and moderate physical activity during gestation to ameliorate mitochondrial dysfunction, thereby alleviating metabolic anomalies in offspring. Promising novel approaches, such as mitochondrial transplantation, represent compelling avenues for future investigations and clinical translation.

In conclusion, this review integrates novel evidence highlighting mitochondrial function as an important factor in the pathophysiology of maternal nutritional imbalances and metabolic disorders across various organ systems in offspring, emphasizing the bidirectional relationship between mitochondrial dysfunction and metabolic dysregulation.

## Figures and Tables

**Table 1:** Maternal nutritional imbalance and offspring organ-specific mitochondrial dysfunction in metabolic defects.

Exposure	Exposure Time Window	Animal Model	Cell/Tissue Type	Alterations in Tissue Mitochondria in the Offspring	Involved Mitochondria Dysfunction	Metabolic Alterations in the Offspring
HFD/HSD/CAF diets [50]	9 weeks before breeding, throughout pregnancy	Wistar rats	Hypothalamus	**Fusion:** ↑ MFN2 (HFD) ↓ DRP1 (CAF)	Mitochondrial fusion	↓ Glucose, leptin, and insulin sensitivity ↑ Fat accumulation; ↑ Insulin resistance ↑ Dyslipidemia
Maternal HFD [51]	6 weeks before mating, throughout gestation and lactation	Sprague–Dawley rats	Hypothalamus	**Mitophagy:** ↑ PINK1 ↑ PARKIN **OXPHOS complexes:** ↓ complexesV	Mitophagy OXPHOS complexes	↑ Body weight ↑ Glucose intolerance ↑ Adiposity ↑ Plasma TG level
Maternal HFD [52]	Throughout pregnancy	Wistar rats	Islet	**Uncoupling Proteins:** ↓ *Ucp2* (Female) **OXPHOS complexes** ↓ ATP synthase (*Atp6*) **Biogenesis:** ↑ *Nrf1* (Female)	Uncoupling Proteins OXPHOS complexes Mitochondrial Biogenesis	Lack of increase in glucose-stimulated insulin secretion and ATP content
Maternal obesogenic diet [53]	6 weeks before breeding, throughout pregnancy	C57BL6/J mice	Placenta	**OXPHOS complexes:** ↑ complex II and ATP synthase (Male) **Biogenesis:** ↑ PGC-1α (Female) **Fission:** ↑ DRP1 (Male) **Uncoupling Proteins:** ↓ UCP2	OXPHOS complexes Mitochondrial Biogenesis Mitochondrial fission Uncoupling Proteins	↑ Rate of small for gestational age ↑ Blood glucose concentrations
Maternal HFD [54]	From gestational day 7	Sprague-Dawley rats	Cardiomyocytes	**Fission:** ↓ MFF **Mitophagy:** ↑ PARKIN ↓ LC3B **OXPHOS complexes:** ↓ complex V (ATP5B)	Mitochondrial fission Mitophagy OXPHOS complexes	↑ Lipid accumulation ↑ Inflammation
Maternal HFD [55]	28 days before breeding	Sprague-Dawley rat	Neonatal rat cardiomyocytes	**Morphology:** shorter, more fragmented mitochondria ↓ fusion and fission event **Respiratory capacity:** ↓ basal OCR **mtDNA copy number:** ↑ mtDNAcn	Altered Morphology Mitochondrial fission Mitochondrial fusion Respiratory capacity	↑ Mean heart-to-body weight ratio ↑ Number and size of lipid droplets in ventricular sections
Maternal HFD [56]	28 days before breeding	Sprague-Dawley rat	Neonatal rat cardiomyocytes	**Morphology:** shorter and wider mitochondria ↓ fusion and fission event ↑ fission–fusion ratios **Fusion:** ↓ MFN1 ↑ MFN2 ↑ OPA1 (Male)	Altered Mitochondrial morphology ↓ Mitochondrial dynamic (pro-fission effect)	↑ Diastolic and systolic dysfunction ↑ Myocardial lipid accumulation
Maternal HFD [57]	28 days before breeding and throughout pregnancy	Sprague-Dawley rats	Heart	**mtDNA copy number:** ↑ mtDNAcn **Respiratory capacity:** ↓ basal oxidation **FAO:** ↓ Endogenous FAO (Male)	Fatty acids oxidation mtDNA copy number respiratory capacity	↑ Risk of mortality ↓ Cardiac and bioenergetic function ↑ Risk of stress induced cardiomyocyte death
Maternal obesity [58]	60 days before conception and throughout pregnancy	Sheep	Fetal heart tissue	**Oxidative stress:** ↓ GSH to GSSG ratio	Oxidative stress	↑ Risk of developing hyperglycemia and hyperinsulinemia ↓ LV mass to heart weight ratio
Maternal HF-LF [59]	during pregnancy	Guangdong Small-ear Spotted pig	Soleus muscle	**Biogenesis:** ↓ PGC-1α ↓ NRF1 **Respiratory enzyme:** ↓ SDH ↓ MDH	Mitochondrial biogenesis respiratory enzyme	↓ Insulin signaling pathway ↓ Glucose tolerance ↑ Glycolytic muscle fibers formation
Maternal GDM (intraperitoneally injected with 100 mg/kg streptozotocin) [60]	on gestational days 6.5 and 12.5	Institute of Cancer Research (ICR) mice	Soleus muscle	**Morphology:** Fewer mitochondria, vacuoles swollen **Biogenesis** ↓ *Pgc-1α*	Altered Mitochondrial morphology Mitochondrial biogenesis fatty acid oxidation	↓ Glucose metabolism Earlier glucose intolerance
Maternal HFD [61]	4 weeks before breeding, throughout pregnancy and lactation	Wistar rats	Soleus muscle	Loss of the characteristic alignment of mitochondria in parallel to myofibrils ↑ mitochondria with low electron density in the matrix, atypical or absent mitochondrial cristae	Altered Mitochondrial morphology	↑ Body weight ↑ Adiposity ↑ Glycemia ↑ Leptinemia
Maternal HFHS [62]	6 weeks before breeding, throughout pregnancy and lactation	C57BL6N mice	Gastrocnemius	↑ Mitochondrial leak respiration ↓ ROS production in red gastrocnemius in response to palmitoyl carnitine (Male) **OXPHOS complexes:** ↓ complex I protein NDUFA9	OXPHOS complexes	↑ Fasting blood glucose ↑ gonadal white adipose tissue
Maternal nutrient excess diet [63]	12 months before pregnancy	Baboons	Fetal soleus muscle	**Biogenesis:** ↓ *PGC-1α* ↓ PGC-1α ↓ *NRF1* **mtDNA copy number** ↓ mtDNAcn **Fusion** ↓ *MFN1*, *MFN2* **Mitochondrial density** ↓ Citrate synthase (CS) **mitochondrial respiratory chain** ↓ cytochrome C protein levels ↓ cytochrome C oxidase subunit I and II transcripts (c*ox1* & c*ox2*) **Sirtuins:** ↓ *SIRT1 & SIRT3*	Mitochondrial biogenesis mitochondrial density mtDNAcn Mitochondria fusion mitochondrial respiratory chain Sirtuins	↑ Total body fat percentage
Maternal HFD [64]	1 week before breeding, throughout pregnancy	heterozygous *PolgA*^D257A^ mutated mice	Gastrocnemius	**Biogenesis:** ↓ PGC-1α **Energy transfer/ETC function:** ↓ VDAC	Energy transfer/ETC function Biogenesis	↑ Weight of adipose tissues ↑ Intramuscular fat accumulation
Maternal HFD [65]	Prepregnancy for 8 weeks	C57BL/6J mice	Gastrocnemius	**mtDNA copy number** ↓ mtDNAcn **Biogenesis:** ↓ *Pgc-1α* ↓ *Tfam*	mtDNA copy number Biogenesis	↓ Glucose tolerance
Maternal Western-style [66]	Prepregnancy for 9 years	Japanese macaques	Fetal muscle	**OXPHOS complexes activity:** ↑ complexe I ↑ complexe IV **Mitochondrial density:** ↓ Citrate synthase (CS) **Uncoupling Proteins:** ↑ *UCP2* ↑ *UCP3* **Sirtuins:** ↑ *SIRT3* **Respiratory capacity:** ↓ Basal oxygen consumption rate (OCR), maximal OCR	OXPHOS complexes Mitochondrial density Uncoupling Proteins Sirtuins respiratory capacity	↓ Muscle oxidative capacity and glucose metabolism
Maternal Fructose diet [67]	60 days before breeding, throughout pregnancy	Dunkin Hartley guinea pigs	Liver	**OXPHOS complexes:** ↑ complexe II, IV (Weaning) ↓ complexe II, IV (Adult) **Energy transfer/ETC function:** ↑ VDAC1 (Weaning) ↓ VDAC1 (Adult)	OXPHOS complexes ETC function	↑ Plasma TG at weaning and adult offspring
Maternal Western diet [68]	8 weeks before breeding, throughout pregnancy and lactation	C57BL/6J mice	Liver	**Biogenesis:** ↓ *Pgc-1α* ↑ *Nrf1* **mtDNAcn:** ↑ mtDNAcn (absence of fibrosis) ↓ mtDNAcn (with fibrosis development) **Fusion:** ↑ *Opa1* (absence of fibrosis) ↓ *Opa1* (with fibrosis development) **Fission:** ↑ *Dnm1l & Dnm2* with fibrosis development **Mitophagy:** ↓ *Bnip3* ↑ *Parkin* (not significant) **OXPHOS complexes:** ↓ *Nd1* ↓ *Cytb & Atp6* (fibrosis) ↓ abundance & activity of complexes III, IV, V	OXPHOS complexes Mitochondrial biogenesis Mitochondrial fission/fusion Mitophagy mtDNA copy number	↑ Body weight gain ↑ Hepatic insulin resistance ↑ Steatosis ↑ Inflammation ↑ Fibrosis
Maternal obesogenic diet [69]	From weaning, throughout pregnancy and lactation	C57BL/6 mice	Liver	**OXPHOS complexes:** ↓ complexes I (NDUFB8), III (UQCRC2), IV (MTCO1), V (ATP5A) ↑ *Uqcrc2 & Atp5a1*	OXPHOS complexes	↑ Body weight ↑ Body-fat content ↑ Circulating TG ↑ Absolute liver weight ↑ Hepatic lipid
Maternal HFHS diet [70]	7 weeks before breeding, throughout pregnancy and lactation	Sprague-Dawley rats	Liver	**Morphology:** ↑ Mitochondria diameter **Fusion:** ↓ *Mfn2* ↑ OPA1 **Mitophagy:** ↑ Parkin	Mitochondrial fusion Mitophagy	↑ Body weight ↑ Hepatic lipid accumulation
Maternal obesogenic diet [71]	Prepregnancy for 60 days	Sheep	Fetal liver	**OXPHOS complexes activity:** ↓ complexes I, II–III and IV ↓ **mitochondrial phospholipid**	OXPHOS complexes activity Mitochondrial phospholipid	NA
Maternal calorie restriction [72]	During pregnancy	Wistar rats	Placenta	**mtDNAcn:** ↑ mtDNAcn **Biogenesis:** ↑ *Pgc-1α* ↑ *Nrf1* ↑ Tfam **Uncoupling Proteins:** ↓ *Ucp2* **Respiratory capability:** ↑ Basal oxygen consumption ↑ Complex I and Complex IV respiratory capacity ↑ Respiratory control ratio (RCR) **ATP content:** **↓** ATP content and ATP/ADP ratio	Biogenesis Respiratory capability	↑ Fetal growth restriction ↓ blood glucose
Maternal nutrient reduction [73]	From gestation day 30 to 90	Baboons	Fetal heart tissue	**OXPHOS complexes:** ↑ complex I (NDUFB8) ↑ complex III (UQCRC2) ↑ complex IV (COXII, COX6C) **Mitochondrial density:** ↓ Citrate synthase (CS)	Mitochondrial density OXPHOS complexes	↑ Risk of cardiovascular disease
Maternal nutrient reduction [74]	From gestation day 39 to 165	Baboons	Fetal heart tissue	**Morphology:** sparse and disarranged cristae dysmorphology **mtDNAcn:** ↑ mtDNAcn **OXPHOS complexes:** ↑ complex I (NDUFB8) ↑ complex III (UQCRC2) **Activity:** ↓ complex I ↓ complex II ↓ complex III **Fission:** ↑ *FIS1* **Mitochondrial density:** ↓ Citrate synthase (CS)	Altered Mitochondrial morphology mtDNAcn OXPHOS complexes Fission Mitochondrial density	Fetal cardiac bioenergetic deficits ↑ lipid peroxidation
Maternal LPD [75]	Throughout pregnancy and lactation	Wistar rats	Liver	**Oxidative stress:** ↑ SOD1 and SOD2 **OXPHOS complexes:** ↓ complex II **Mitochondrial density:** ↓ citrate synthase **Biogenesis** ↓ TFAM	Oxidative stress OXPHOS complexes Mitochondrial density Biogenesis	↓ Aerobic metabolism
Maternal LPD [76]	From Day 4 of the pregnancy to delivery	Wistar rats	Gastrocnemius (Male)	**Fusion:** ↓ *Mfn1 & Mfn2* **Fission:** ↓ *Fis1* **Biogenesis:** ↓ *Pgc-1β*, *Nrf1*, *and Esrra* **Respiratory capability:** ↓ the ATP-linked oxygen consumption rate (OCR), maximal, spare respiratory, non-mitochondrial respiration-associated OCRs **OXPHOS complexes:** ↓ complex I (*Ndufb8*) **mitochondrial pyruvate transport and metabolism:** ↓ *Mpc1 & Pdha1* **mitochondrial fatty acid transporters:** ↑ *Cpt1a* and *Cpt2*	Mitochondrial biogenesis Mitochondrial fission/fusion Mitophagy Respiratory capability OXPHOS complexes mitochondrial pyruvate transport and metabolism mitochondrial fatty acid transporters	glucose intolerant and insulin resistant
Maternal GFR [52]	Throughout pregnancy	Wistar rats	Islets	**Biogenesis:** ↑ *Pgc-1α* (Female) **↓** *Tfam* (Female) **Uncoupling Proteins:** ↓ UCP2 **OXPHOS complexes** ↓ ATP synthase (*Atp6*)	Mitochondrial biogenesis Uncoupling Proteins OXPHOS complexes	Lack of increase in glucose-stimulated insulin secretion and ATP content
Maternal LPD [77]	For 2 months after weaning	Wistar rats	Islet	**Oxidative stress:** ↑ 8-oxo-dG DNA content (F2 offspring of malnourished F1 females)	Oxidative stress	↑ FBG ↓ OGTT
Maternal PR diet [78]	Gestation (E0 to E17)	Sprague-Dawley rats	Fetal hypothalamus	**OXPHOS complexes:** ↑ Genes encoding mitochondrial respiratory chain proteins ↑ Complex II (SDHB), III (UQCRC2), IV (MTCO1), V (ATP5A) **Membrane permeability:** ↑ Mitochondrial membrane potential	OXPHOS complexes Mitochondrial membrane permeability	↑ Neuronal-committed progenitors Potential long-term impaired hypothalamic function & metabolic disorders

HFD: high-fat diet; HSD: high sucrose diet; CAF: cafeteria diet; GDM: gestational diabetes mellitus; HFLF: high fat, low fiber; HFHS: high fat, high sucrose; GFR: global food restriction; LP: low protein; PR: protein restriction; MFN2: mitofusin 2; Dnm1l/DRP1: dynamin-related protein 1; PINK1: PTEN induced kinase 1; UCP2: uncoupling protein 2; ATP6: ATP synthase membrane subunit 6; NRF1: nuclear respiratory factor 1; PGC-1α: peroxisome proliferator-activated receptor-γ coactivator 1α; MFF: mitochondrial fission factor; LC3B: microtubule-associated protein 1A/1B-light chain 3 beta; ATP5B: ATP synthase F1 subunit beta; OCR: oxygen consumption rate; mtDNAcn: mitochondrial DNA copy number; OPA1: optic atrophy 1; FAO: fatty acid oxidation; SDH: succinate dehydrogenase; MDH: malate dehydrogenase; NDUFA9: NADH dehydrogenase 1 alpha subcomplex, 9; CS: citrate synthase; Cox1: cytochrome c oxidase subunit I; Cox2: cytochrome c oxidase subunit II; SIRT1: sirtuin 1; SIRT3: sirtuin 3; VDAC: voltage-dependent anion channel; Tfam: mitochondrial transcription factor a; Dnm2: dynamin 2; Bnip3: bcl2-interacting protein 3; Nd1: NADH dehydrogenase subunit 1; Cytb: cytochrome b; NDUFB8: NADH dehydrogenase 1 beta subcomplex subunit 8; UQCRC2: ubiquinol-cytochrome c reductase core protein 2; MTCO1: mitochondrially encoded cytochrome c oxidase I; FIS1: fission, mitochondrial 1; PGC-1β: peroxisome proliferator-activated receptor-γ coactivator 1β; Esrra: estrogen-related receptor alpha; MPC1: mitochondrial pyruvate carrier 1; PDHA1: pyruvate dehydrogenase e1 alpha 1 subunit; CPT1a: carnitine palmitoyltransferase 1a; CPT2: carnitine palmitoyltransferase 2; SDHB: succinate dehydrogenase complex iron sulfur subunit b; TG: triglyceride; ATP: adenosine triphosphate; FBG: fasting blood glucose; OGTT: oral glucose tolerance test. Mark explanations: upward (↑) and downward (↓) arrows indicate an increase (or upregulation) and a decrease (or downregulation) in the respective processes or levels.

## Data Availability

No new data were created or analyzed in this study. Data sharing is not applicable to this article.

## Abbreviations

The following abbreviations are used in this manuscript:

AgRP	Agouti-Related Peptide
AMPK	AMP-activated Protein Kinase
ATP	Adenosine Triphosphate
ATP5A	ATP Synthase Subunit Alpha, Mitochondrial
ATP5B	ATP Synthase Subunit Beta, Mitochondrial
ATP6	ATP Synthase Membrane Subunit 6
BNIP3	BCL2-Interacting Protein 3
CAF	Cafeteria Diet
CPT1/2	Carnitine Palmitoyltransferase 1/2
CREB	cAMP-Response Element Binding Protein
CS	Citrate Synthase
COX	Cytochrome C Oxidase
Cox1	Cytochrome C Oxidase Subunit I
Cox2	Cytochrome C Oxidase Subunit II
Cytb	Cytochrome b
DNM1L	Dynamin 1 Like
Dnm2	Dynamin 2
DOHaD	Developmental Origins of Health and Disease
DRP1	Dynamin-Related Protein 1
E0 to E17	Embryonic Day 0 to 17
ETC	Electron Transport Chain
Esrra	Estrogen-Related Receptor Alpha
FBG	Fasting Blood Glucose
FAO	Fatty Acid Oxidation
FGF21	Fibroblast Growth Factor 21
Fis1	Mitochondrial Fission 1 Protein
GDM	Gestational Diabetes Mellitus
GLUT4	Glucose Transporter Type 4
GSIS	Glucose-Stimulated Insulin Secretion
GSH	Glutathione
GWG	Gestational Weight Gain
GFR	Global Food Restriction
HFD	High-Fat Diet
HFLF	High Fat, Low Fiber
HFHS	High Fat, High Sucrose
HSD	High Sucrose Diet
IDH2	Isocitrate Dehydrogenase 2
IUGR	Intrauterine Growth Restriction
LC3B	Microtubule-Associated Protein 1A/1B-Light Chain 3 Beta
LDL-c	Low-Density Lipoprotein Cholesterol
LP	Low Protein
LPD	Low-Protein Diet
MAFLD	Metabolic Dysfunction-Associated Fatty Liver Disease
MFN	Mitofusin
MFF	Mitochondrial Fission Factor
MPC1	Mitochondrial Pyruvate Carrier 1
mROS	Mitochondrial Reactive Oxygen Species
MSC	Mesenchymal Stem Cell
MQC	Mitochondrial Quality Control
MTCO1	Mitochondrially Encoded Cytochrome C Oxidase I
mtDNA	Mitochondrial DNA
mtDNAcn	Mitochondrial DNA Copy Number
NDUFA9	NADH Dehydrogenase 1 Alpha Subcomplex, 9
NDUFB8	NADH Dehydrogenase 1 Beta Subcomplex Subunit 8
ND1	NADH Dehydrogenase Subunit 1
NMN	Nicotinamide Mononucleotide
NRF1	Nuclear Respiratory Factor 1
OGTT	Oral Glucose Tolerance Test
OPA1	Optic Atrophy 1
OXPHOS	Oxidative Phosphorylation
PGC-1α	Peroxisome Proliferator-Activated Receptor-γ Coactivator 1α
PGC-1β	Peroxisome Proliferator-Activated Receptor-γ coactivator 1β
PDHA1	Pyruvate Dehydrogenase E1 Alpha 1 Subunit
PINK1	PTEN Induced Kinase 1
POMC	Pro-Opiomelanocortin
PolG	Polymerase γ
PPARα	Peroxisome Proliferator-Activated Receptor Alpha
PR	Protein Restriction
RyR2	Ryanodine Receptor 2
SIRT	Sirtuin
SDH	Succinate Dehydrogenase
SDHB	Succinate Dehydrogenase Complex Iron Sulfur Subunit B
SGA	Small for Gestational Age
SOD	Superoxide Dismutase
TFAM	Transcription Factor A, Mitochondria
TG	Triglyceride
UCP	Uncoupling Protein
UQCRC2	Ubiquinol-Cytochrome C Reductase Core Protein 2
VDAC	Voltage-Dependent Anion Channel
WD	Western Diet
